# Treatment Response to Acute Total Ophthalmoplegia in Primary Pituitary Lymphoma: A Case Report and Review of the Literature

**DOI:** 10.7759/cureus.51478

**Published:** 2024-01-01

**Authors:** Saygı Uygur, Derya Karataş, Gözde Arslan, Ahmet Dağtekin, Emel Avcı

**Affiliations:** 1 Neurosurgery, Mersin University School of Medicine, Mersin, TUR; 2 Pathology, Mersin University School of Medicine, Mersin, TUR

**Keywords:** surgical resection, chemotherapy, sellar-parasellar lesion, ophthalmoplegia, primary pituitary lymphoma

## Abstract

Primary pituitary lymphoma (PPL) is an extremely rare localized lymphoma without systemic involvement. The most common clinical presentations of PPL are hypopituitarism, headaches, and ophthalmoplegia. Diagnosing PPL without a biopsy is almost impossible. There is no study that has specifically investigated and reviewed treatment responses to the ophthalmological symptoms of PPL patients. Herein, we present a 66-year-old female patient who had acute-onset total ophthalmoplegia and headache as admission symptoms, which was diagnosed as PPL after subtotal resection. In the present study, we discussed the response of ophthalmological symptoms to treatment with a review of the literature. Only 18 reported cases had postoperative ophthalmological examination, and in 94.4% of these cases, ophthalmoplegia resolves after surgery and chemotherapy. The complete resection rate of the PPL in the literature was found to be as low as 12.3% in this region because of the hard and adhesive nature of the tumor. Our review unveiled that complete recovery of ophthalmoplegia can be achieved even in the late phase of the symptoms. In the present case, ophthalmoplegia resolved completely following subtotal resection and rituximab, high-dose methotrexate, and cytarabine treatment.

## Introduction

Primary pituitary lymphoma (PPL) is a localized lymphoma in the pituitary gland, which lacks systemic involvement [1-18]. It is an extremely rare tumor, with only 57 reported cases in English literature to the best of our knowledge. The pituitary gland is a common location for primary intracranial tumors (15%) as well as systemic metastases. The most common tumors of the sellar and parasellar region are adenomas (90%) followed by meningiomas, craniopharyngiomas, germ cell tumors, gliomas, and metastases [10]. Even though magnetic resonance imaging (MRI) and positron emission tomography-computed tomography (PET-CT) could be useful for differential diagnosis and exclusion of systemic involvement, diagnosing PPL without a biopsy is almost impossible owing to the nonspecific nature of its history, symptoms, laboratory investigation, and radiological findings [14,19]. Ophthalmoplegia is one of the most common severe neurological symptoms in sellar and parasellar lesions. However, no study has specifically investigated and reviewed treatment responses to ophthalmological symptoms of PPL patients. Treatment options after surgical resection include chemotherapy/high-dose methotrexate with or without radiotherapy. To our knowledge, there are 34 PPL cases with ophthalmologic symptoms, and only 18 of them had pre- and postoperative ophthalmological examinations.

In this report, we present the case of a 66-year-old female patient who presented with acute total ophthalmoplegia of the left eye and severe headache. Initially, lymphocytic hypophysitis was suspected by a multidisciplinary team (which consisted of a neuroradiologist, neurologist, and endocrinologist) and treated with steroids. However, upon resection of the lesion, histopathological findings indicated lymphoma. At the two-month follow-up, the lesion was almost completely resolved in the MRI, and the patient's ophthalmoplegia and headache had resolved completely. At the four-month follow-up, she was symptom-free and had normal endocrinological function. In this article, we present the first PPL case with acute-onset total ophthalmoplegia and its response to treatment with a review of the literature.

## Case presentation

**Figure 1:**
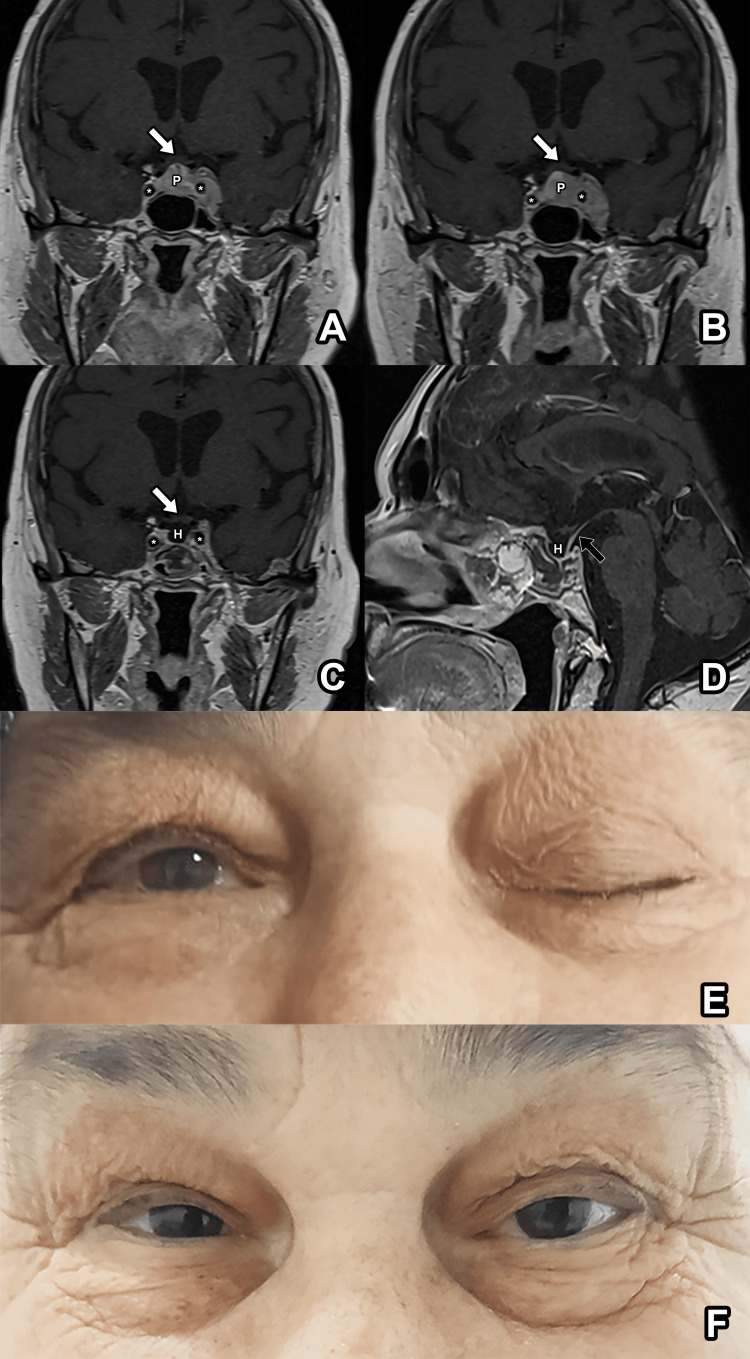
Pre- and postoperative MRI and ophthalmological examination of the patient. A) Initial coronal T1-weighted contrast-enhanced magnetic resonance imaging (MRI) scan reveals Knosp grade 4 pituitary lesion. B) Coronal T1-weighted contrast-enhanced MRI scan obtained a month after the initial MRI reveals that the tumor significantly increased in size (from 12.4 x 28.2 mm to 14.2 x 31.6 mm). Coronal (C) and sagittal (D) T1-weighted contrast-enhanced MRI scans obtained in the second postoperative month revealed almost complete disappearance of the tumor. E) Preoperative ophthalmological examination reveals total ophthalmoplegia, which completely resolved (F) in the second postoperative month. White arrow: optic chiasm, P: primary pituitary lymphoma, asterisk: internal carotid artery, H: hypophyseal fossa, black arrow: pituitary stalk.

A 66-year-old female with previously known diabetes mellitus and hypertension that are under control initially presented to another hospital with a three-day history of severe headache, double vision, progressive drooping of the left eyelid, and loss of postoperative movement in the left eye. These symptoms persisted for a month until she eventually sought treatment at our hospital. A neurological examination revealed total ophthalmoplegia, accompanied by a loss of visual acuity in the left eye and mild hypoesthesia on the left side of her face (Figure 1E).

A subsequent MRI scan revealed a pituitary lesion with T1 and T2 isointensities and contrast enhancement. Notably, a complete invasion of the left cavernous sinus was observed, classified as Knosp grade 4 (Figure 1A). Laboratory test results indicated panhypopituitarism without diabetes insipidus. PET-CT scan revealed increased activity in the sellar region without systemic uptake. Initially, the patient received a preliminary diagnosis of lymphocytic hypophysitis by a multidisciplinary team (which consisted of a neuroradiologist, neurologist, and endocrinologist) and underwent 80 mg per day methylprednisolone treatment in the endocrinology department.

Despite a month of steroid treatment, no improvement was observed in the patient’s headache and ophthalmoplegia. A follow-up MRI scan revealed that the lesion had increased in size (from 12.4 x 28.2 mm to 14.2 x 31.6 mm) (Figure 1B). Subsequently, the patient underwent endoscopic transsphenoidal surgery, revealing a hard, grey, amorphous mass, invading the sphenoid bone. Partial resection was performed owing to the adherent and hard nature of the tumor. During the surgery, two samples were obtained for biopsies: one from the pituitary gland and the other from the posterior wall of the sphenoid sinus bone.

**Figure 2:**
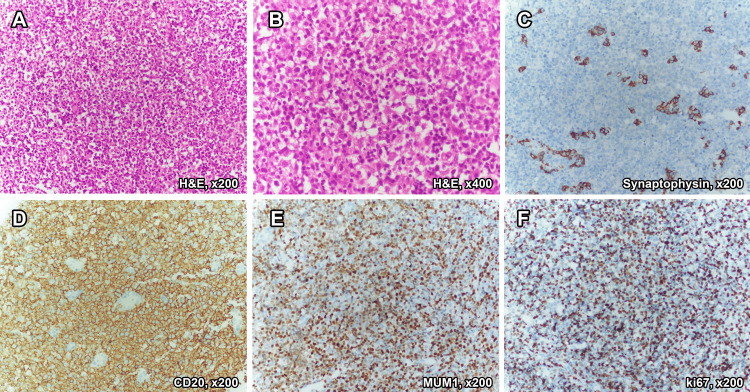
Pathological specimen. (A,B) Biopsy sample obtained from the pituitary gland shows diffuse atypical lymphoid infiltration (hemotoxylin and eosin, ×200; ×400). (C) Residual pituitary cells stain positively for synaptophysin (×200). (D,E) Immunohistochemical examination reveals positive staining for CD20 and MUM1 in the lymphoid cells (×200). (F) Proliferative activity of 80% is observed with Ki67 (×200).

Histopathological examination of the biopsy specimen obtained from the pituitary gland revealed diffuse infiltration of atypical lymphoid cells among the residual pituitary cells. The tumor cells stained positively for CD20, LCA, Bcl-2, and MUM1 and negatively for pancytokeratin, CD10, desmin, GFAP, and S100. The positive MUM1 and negative CD10 staining indicated a nongerminal matrix lymphoma. Moreover, the Ki-67 proliferation index was 80%. Consequently, a diagnosis of diffuse large B-cell lymphoma was made. Additionally, lymphoid cell infiltration was observed between the bony trabeculae (Figure 2).

The severe headache of the patient resolved immediately after the surgery. Treatment was initiated with rituximab, high-dose methotrexate, and cytarabine. At the two-month follow-up, an MRI scan revealed a near-complete resolution of the tumor (Figures 1C-1D). The patient’s ophthalmoplegia and headache had resolved completely at the two-month follow-up, and at the four-month follow-up, she was symptom-free and had normal endocrinological function (Figure 1F).

## Discussion

PPL is an exceptionally rare disease, and overlapping findings on MRI scans with other pituitary lesions pose diagnostic challenges. Both PPL and adenomas may exhibit increased uptake during a PET-CT scan, further complicating the differential diagnosis. Diagnosing PPL often necessitates surgical biopsy, which is the gold standard for a definitive diagnosis [14]. Imaging with PET-CT is also important to exclude a systemic lymphoma with central nervous system (CNS) metastasis to conclude a PPL diagnosis [19]. According to their comprehensive genomic analysis, Kimbrough et al. suggested that PPL should be considered a distinct entity from a primary central nervous system lymphoma (PCNSL) because MYD88, CDKN2A, or CD79A alterations, which are commonly found in PCNSL, were not apparent in PPL [19]. PCNSL can shrink or resolve completely with steroid treatment, resulting in the vanishing tumor phenomenon. However, the rate of complete and incomplete resolution in response to steroid treatment is 15% and 25%, respectively, in PCNSL [20]. Despite ongoing steroid treatment in our case, consecutive MRI scans obtained within one month indicated an aggressive growth pattern. This rapid growth pattern and persistent symptoms were suggestive of malignant lesions. The diagnosis of PPL could not be established despite the use of MRI and PET-CT preoperatively.

The most common clinical presentations of PPL are hypopituitarism (75%) and headaches (57.5%) [14]. Partial and total ophthalmoplegia are one of the severe neurological deficits caused by PPL. However, whether or how much these symptoms will improve is still a concerning issue in this malignant disease. In the literature, only 57 PPL cases have been reported, and 34 of these cases had ophthalmological symptoms. Only 18 of these reported cases included pre- and postoperative ophthalmological examination (Table 1) [1-18]. This is the first study to investigate and review treatment responses to the ophthalmological symptoms of PPL patients. Among the 18 investigated patients, ophthalmoplegia resolved completely in 10 (55.5%) of them, whereas it resolved partially in seven (38.9%) and did not resolve in one (5.6%) of them. A paucity of studies makes it challenging to reach reliable data on symptom improvement after treatment. Our review of the literature revealed that 94.4% of the PPL cases’ ophthalmoplegia resolves after surgery and chemotherapy. Additionally, this review unveiled that complete recovery of ophthalmoplegia can be achieved even in the late phase of the symptoms. Similar to the literature, our patient’s total ophthalmoplegia completely resolved, after two months of the initial symptoms, with subtotal resection and rituximab, high-dose methotrexate, and cytarabine treatment. At the four-month follow-up, our patient was symptom-free and had normal endocrinological function.

**Table 1:** Summary of clinical results of 18 cases and present case. M: Male, F: Female, STR: Subtotal resection, n/a: Not available [1-18].

Authors and year	Gender/age	Cranial nerve deficit	Resection	Initial symptoms to treatment	Resolution
Shaw et al., 1997 [1]	F 73	VI	Biopsy	Two weeks	Partial
Kuhn et al., 1999 [2]	F 67	II, III	STR	Acute	Partial
Lee et al., 2002 [3]	M 42	II	Resection	Three months	Complete
Katz et al., 2003 [4]	F 64	VI	Resection	Acute	Complete
Rudnik et al., 2007 [5]	M 73	II, III	STR	n/a	Partial
Quintero Wolfe et al., 2009 [6]	F 45	II, III, V	Resection	Four days	Complete
Martinez et al., 2011 [7]	F 71	II	Resection	Several weeks	Complete
Carrasco et al., 2012 [8]	F 49	VI	Biopsy	Acute	Complete
Rainsbury et al., 2012 [9]	F 67	II	Resection	Acute	Complete
Tarabay et al., 2016 [10]	M 73	II, III	STR	n/a	Partial
Ban et al., 2017 [11]	M 74	III	Resection	Several weeks	Partial
Velho et al., 2018 [12]	F 55	II	Resection	n/a	Complete
Harjanto et al., 2020 [13]	F 75	III	Biopsy	One month	Complete
Duan et al., 2021 [14]	M 61	II, III	Biopsy	14 months	Complete
Zhang et al., 2021 [15]	M 61	II, III	Biopsy	Four months	Partial
Abdelaziz et al., 2022 [16]	F 49	II	Biopsy	Acute	Partial
Seferi et al., 2023 [17]	M 31	II, III	Resection	Three days	No resolution
Yoshida et al., 2023 [18]	M 78	II	Resection	n/a	Complete
Present case	F 66	II, III, IV, VI	STR	Two months	Complete

In the literature, only seven (12.3%) cases of complete resection in PPL have been reported. Owing to the hard and adhesive nature of the tumor, achieving complete resection is usually not feasible similar to our case. Duan et al. reported that PPL might invade the sphenoidal sinus bone and mucosa. They suggested that a biopsy of the sinus mucosa could decrease the risk of surgery while making a diagnosis [14]. Similarly in our case, after subtotal tumor resection, a biopsy has been taken from the sphenoid bone trabeculae, which consisted of lymphoid cell infiltration. The severe headache of our patient resolved immediately after subtotal resection of the tumor and before chemotherapy treatment. 

Most reported cases of PPL are treated with the same regimen as that for PCNSL [10]. Treatment options after surgical resection include chemotherapy with or without radiotherapy [14,19]. Kimbrough et al. reported that PPL should be treated differently from PCNSL and suggested that an anatomy-oriented approach that includes both CNS-penetrating and non-CNS penetrating agents should be implemented. Recent studies have shown promising outcomes in PCNSL with the inclusion of high-dose methotrexate in the chemotherapy regimen [19]. However, in the present study, a 20.6% mortality rate within six months has been found among 34 PPL patients with ophthalmological symptoms. In our patient, steroid treatment did not affect symptoms or tumor regression; in contrast, cranial nerve deficits that were apparent for more than two months completely resolved with subtotal resection and rituximab, high-dose methotrexate, and cytarabine treatment.

## Conclusions

PPL is an extremely rare, rapidly growing, and aggressive tumor and should be considered in the differential diagnosis of pituitary lesions with total ophthalmoplegia. A definitive diagnosis usually cannot be made with only radiological imaging and clinical presentations. The golden standard for the definitive diagnosis is surgical biopsy. Steroid treatment may not affect radiological progression or alleviate the symptoms of PPL. It is demonstrated that despite the tumor’s aggressive nature and delayed histopathological diagnosis, favorable ophthalmological outcomes could be achieved even with subtotal surgical resection and chemotherapy/high-dose methotrexate.
